# Clinical Insights and Dermatological Recommendations for Non-Melanoma Skin Cancers (NMSCs) in Long-Term Hydroxyurea (HU) Therapy

**DOI:** 10.7759/cureus.57133

**Published:** 2024-03-28

**Authors:** Carmen Iliescu, Cristina Beiu, Iulian Slavu, Andreea Racoviță, Cristina Orlov Slavu

**Affiliations:** 1 Dermatology, Elias Emergency University Hospital, Carol Davila University of Medicine and Pharmacy, Bucharest, ROU; 2 Oncologic Dermatology, Elias Emergency University Hospital, Carol Davila University of Medicine and Pharmacy, Bucharest, ROU; 3 General Surgery, “Prof. Dr. Agrippa Ionescu” Emergency Clinical Hospital, Bucharest, ROU; 4 Oncology, Elias Emergency University Hospital, Carol Davila University of Medicine and Pharmacy, Bucharest, ROU

**Keywords:** myeloproliferative disorders, squamous cell carcinoma, skin toxicity, hydroxyurea, non-melanoma skin cancer

## Abstract

Hydroxyurea (HU), an anti-metabolite ribonucleotide reductase inhibitor, is commonly used to treat several myeloproliferative disorders, including polycythemia vera. However, patients receiving long-term treatment with HU may experience a variety of cutaneous side effects, with non-melanoma skin cancers (NMSCs) emerging as the most challenging and destructive. HU-induced carcinogenesis can be attributed to both the drug's mutagenic potential and impaired DNA repair following damage by external triggers such as ultraviolet light. We report a unique case of multiple aggressive NMSCs distributed within sun-exposed areas in an 81-year-old woman receiving chronic therapy with HU for 15 years. The case draws the clinician’s attention to the increased incidence of NMSCs in this population and highlights the need for regular dermatologic monitoring. We also elaborate relevant insights and recommendations to assist healthcare providers in managing HU-related NMSCs development and progression.

## Introduction

Non-melanoma skin cancers (NMSCs) represent the most frequently diagnosed malignancies within Caucasian populations, demonstrating a rising global incidence trend [1]. While these cancers are generally associated with low mortality rates, neglected tumors, particularly squamous cell carcinomas (SCCs), may pose life-threatening risks due to local infiltration or metastasis [1]. 

Common risk factors for NMSCs often include exposure to ultraviolet (UV) light, advanced age, light skin pigmentation, immunosuppressive therapy, chronic inflammation and long-term therapy with photosensitizing medications [2].

Hydroxyurea (HU) is an antineoplastic agent that inhibits the enzyme ribonucleoside reductase and stands as a cornerstone in the therapeutic arsenal for managing myeloproliferative disorders. However, its extensive use brings to light a range of cutaneous side effects, with NMSCs emerging as a particularly concerning consequence [3]. Although there are limited reports of NMSCs cases following HU therapy, recent years have witnessed a growing body of evidence on this subject, emphasizing the critical role of dermatological monitoring [3].

We report the case of a 81-year-old patient who developed multiple NMSCs while on long-term therapy with HU for polycythemia vera (PV), underscoring the importance of increased awareness surrounding the potential dermatological toxicity associated with HU therapy.

## Case presentation

**Figure 1:**
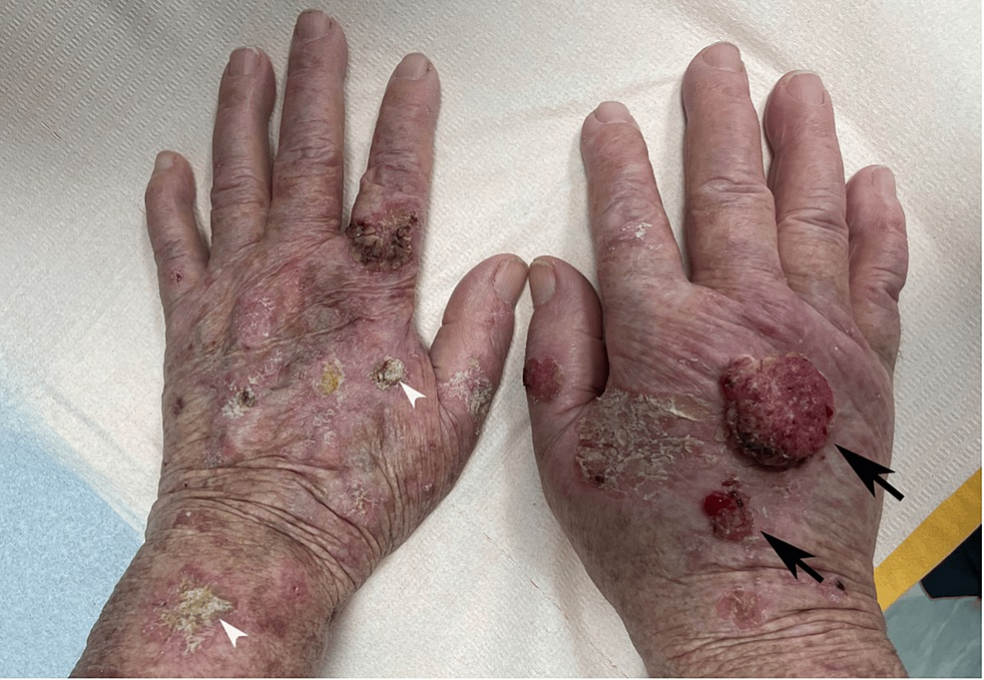
Clinical aspects of the lesions Two moist, eroded, and friable exophytic tumors, measuring 5 cm and 2.5 cm, respectively, in greatest diameter (black arrows), as well as multiple hyperkeratotic and infiltrated erythematous plaques (white arrowheads) can be noticed on the background of heavily sun-damaged skin on the dorsal hands and forearms.

**Figure 2:**
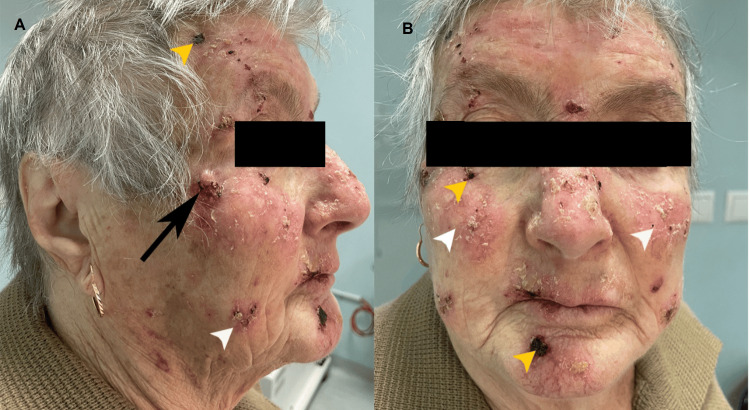
Clinical aspects of the lesions A large nodule (black arrow) with rolled borders and central ulceration located within the right zygomatic area (A). Numerous scaly erythematous plaques (white arrowheads), as well as multiple crusted eroded and ulcerated lesions (yellow arrowheads) arising in sun-damaged skin of the face (B).

An 81-year-old woman with fair phenotypic features (Fitzpatrick type I) and a 15-year history of HU therapy up to 4000 mg daily for PV was referred to our Dermatology Department in December 2023 for the management of progressively evolving skin lesions within photo-distributed areas, such as the face, forearms and dorsal aspect of the hands. Physical examination revealed the presence of two large, exophytic tumors, located on the dorsal side of the right hand (Figure 1) as well as an ulcerated nodule on the right zygomatic area (Figure 2A). The patient had evidence of extensive actinic damage and also presented multiple erythematous scaling plaques and hyperkeratotic papules, some of which ulcerated, involving not only the face, but also both forearms and hands (Figures 1, 2).

From the patient's medical history, the following details have been documented: she has been engaged as an outdoor worker in a rural area of Romania for her entire life, and the onset of widespread cutaneous lesions began over a decade ago as simple erythematous patches and plaques that did not cause any discomfort. It was not until 2019 that the patient sought dermatological consultation at a different dermatological center, when a problematic ulcerated nodular lesion appeared on the right cheek. This lesion extended to the free border of the right lower eyelid and required excision, revealing it to be an ulcerated, nodular, and infiltrative basal cell carcinoma (BCC). At that point, a hypothesis was postulated suggesting that the extensive cutaneous manifestations could be attributed to HU toxicity, given that the patient had been undergoing HU therapy for over a decade.

Consequently, the hematologist discontinued the HU treatment and initiated a six-month course of interferon therapy, which was subsequently interrupted due to the patient's intolerance. The patient's HU treatment was reinstated, and in 2022, an additional therapeutic measure involved the introduction of ruxolitinib at a daily dosage of 20 mg. It's important to note that even after the diagnosis of BCC in 2019, no dermatological interventions were initiated for the numerous additional cutaneous lesions. Moreover, the patient did not undergo regular dermatological surveillance until her presentation at our clinic in December 2023.

Considering the patient’s medical history and clinical aspect of the lesions, we suspected the development of numerous, extensive, drug-induced basal and squamous cell carcinomas. A 5-mm punch biopsy of one of the exophytic tumors was performed. Histopathological analysis of the samples showcased the presence of moderately differentiated SCC cells on hematoxylin and eosin staining. Additional immunohistochemical analysis revealed that the tumor cells tested positive for p53 and epithelial membrane antigen (EMA) while testing negative for p16.

The patient also underwent comprehensive computed tomography and magnetic resonance imaging which revealed no significant changes. A hematological reevaluation was also recommended to determine whether the medicine may be withdrawn and if there are any alternatives available.

Taking into account the presence of numerous skin cancerous lesions at various locations, the patient was presented with a range of therapeutic alternatives. These included conventional surgical intervention for the most sizable tumors, non-surgical modalities like radiation therapy and cryosurgery for lesions where surgical intervention was not feasible or preferred, as well as field-directed treatments such as photodynamic therapy (PDT) or treatment with topical agents such as imiquimod or 5-fluorouracil (5-FU). The patient opted exclusively for surgical treatment in the case of the three ulcerating tumors. The results from the pathological examinations indicated that the two observed lesions located on the dorsum of the hands were moderately differentiated SCCs, whereas the lesion in the zygomatic area was identified as a BCC.

She deferred treatment for the remaining lesions, citing her inability to endure the expected local skin reactions. The patient also received counseling regarding the importance of reducing UV exposure and consistently employing UV protection measures.

## Discussion

We presented a case of multiple highly aggressive NMSCs in a patient undergoing chronic anti-myeloproliferative therapy with HU for 15 years. We additionally want to emphasize the critical importance of routine skin examinations in this population.

Despite its known ease of use, effectiveness, and general tolerance, long-term HU therapy has led to numerous reports highlighting the occurrence of cutaneous complications. These adverse cutaneous reactions encompass a wide spectrum of manifestations, ranging from alopecia, hyperpigmentation, melanonychia, xerosis, palmar or plantar keratoderma, to chronic nonhealing ulcers, dermatomyositis-like eruptions, actinic keratosis, as well as various types of malignancies, with cutaneous SCC emerging as one of the most feared effects [4].

In a systematic review conducted in 2021, which examined six observational studies and four traditional reviews, a significant association was identified between the occurrence of NMSCs and the use of HU. Among the reviewed studies, eight out of 10 reported such an association [3]. These studies identified potential risk factors for NMSCs in HU-treated individuals, including advanced age, prolonged exposure to sunlight, higher dosages of HU, and an extended duration of therapy. The patient in the current report matched all the above risk factors: 81-year-old female, with cumulative exposure to solar radiation, and high dosages of HU for 15 years. Additionally, she presented skin phototype Fitzpatrick I - fair complexion, blue eyes, and blond natural hair, which matches the classic demographical profile seen in patients with cutaneous malignancies.

**Figure 3:**
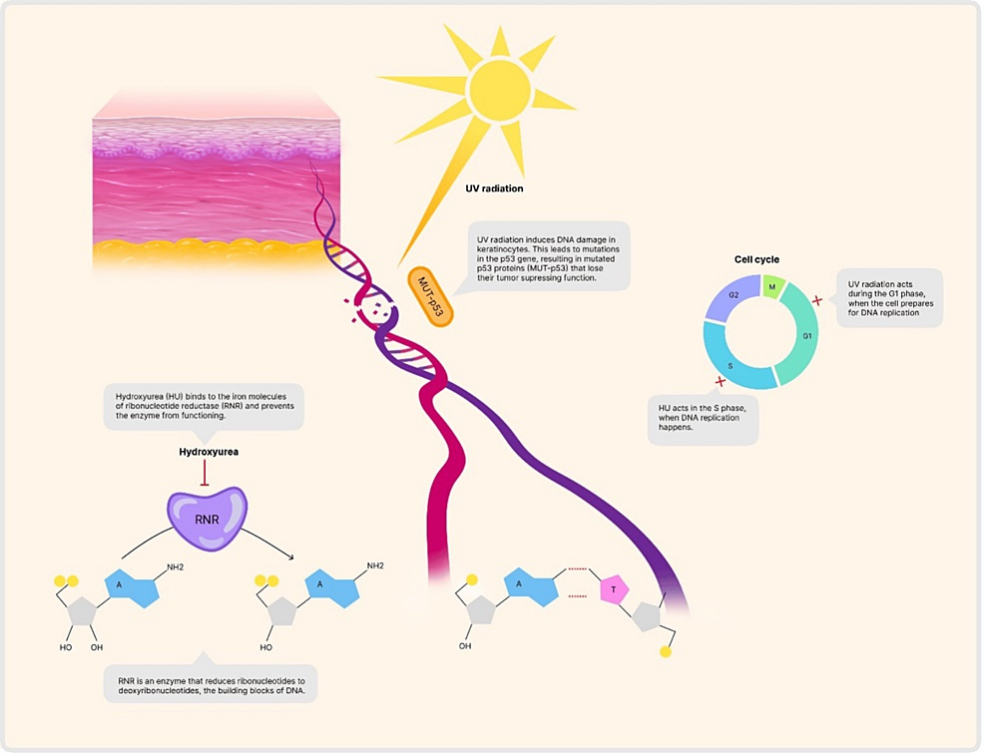
Pathogenesis of HU-associated squamous dysplasia HU and UV radiation act concurrently in promoting the expansion of p-53-clonal keratinocytes. HU: hydroxiurea; UV: ultra violet; RNR: ribonucleotide reductase Image Credit: Andreea Racoviță

HU inhibits DNA synthesis by blocking down the S phase of the cell cycle and induces a damaged DNA repair in epithelial tissues, further sensitizing basal keratinocytes against UV irradiation [5]. As a result, HU and UV-radiation act concurrently in promoting the expansion of p-53 clonal keratinocytes in epithelial dysplasia especially in photo-distributed areas [6]. These findings are aligned with the current case, as all lesions developed within sun-exposed areas over a relatively short period. Moreover, another interesting fact was the presence of p-53 up-regulation on immunohistochemical staining, as seen in the so-called "squamous dysplastic” process induced by HU (Figure 3) [7].

In terms of chronology, NMSCs may develop with a latency period ranging from months to several years after initiation of HU treatment [6]. Thus, patients receiving this type of medicine should undertake periodical dermatology examinations. Similar to individuals using immunosuppressive medications, full-body skin checks are recommended every three to 12 months, for an extended period [8]. Any signs of cutaneous toxicities related to treatment should be acknowledged, notably, any NMSCs. This way, clinicians will be more diligent in addressing these scenarios, with a more timely evaluation of potential subclinical tumor expansion. In our case, the patient did not benefit from these regular skin exams, presenting with a remarkably extensive clinical eruption.

The patient in the current report was also started on ruxolitinib 18 months ago, which, according to the existing literature, may additionally induce skin cancer progression. ruxolitinib alters the number and function of T-lymphocytes and NK-cells, by blocking Janus kinase 1 and 2 receptors, further promoting an imbalance in immune and inflammatory responses [9]. When Ruxolitinib treatment was initiated, the patient had received a BCC diagnosis two years prior and reported pre-existing multiple skin lesions. The patient did not attribute the progression of these lesions to the administration of Ruxolitinib. Given the absence of ongoing dermatological monitoring, it is challenging to definitively ascertain whether Ruxolitinib had a cumulative exacerbating effect in conjunction with HU.

Therapeutic strategies for skin malignancies in this category of patients are consistent with general recommendations for NMSC with few notable particularities. On the one hand, current literature data highlights the importance of HU therapy cessation to achieve complete tumor eradication. If this approach is not feasible, a low dose of oral retinoids, as chemopreventative medication, may be added to the therapy regimen [7]. In our case, discontinuation of HU therapy was not feasible, and the initiation of a low oral retinoid dose was not instituted due to conflicting literature regarding the potential advantages of using oral retinoids for NMSC chemoprevention in individuals receiving HU treatment, with a lack of clear regulatory guidelines in this regard [7].

As for the other dermatological therapeutic alternatives, they include both surgical and non-surgical interventions (radiation therapy, cryosurgery, PDT, topical medications) [2]. However, when the disease has advanced significantly, the radical treatment of these NMSCs is often not possible and the above-mentioned methods involve substantial morbidity that may frequently outweigh potential benefits, particularly in elderly patients with limited life expectancies.

Once again we underscore the significance of early diagnosis, particularly in this population of elderly individuals who are already suffering from other primary neoplastic diseases and multiple comorbidities. Surgery may not be a viable option for many of these patients, and some may find it challenging to tolerate the anticipated local skin reactions or discomfort associated with various non-surgical interventions [2]. Moreover, the probability of achieving a successful outcome with delayed treatments of these skin cancers in advanced stages can vary significantly. Hence, many patients, as exemplified in our case, may opt to defer treatment options in advanced disease stages.

Similar to patients undergoing immunosuppressive treatment and organ transplant recipients, patients receiving HU therapy also constitute a high-risk population [10]. However, unlike these groups, there are currently no established recommendations for preventive strategies aimed at reducing the considerable and increasing burden of cutaneous SCC in HU-treated patients.

Consequently, we have formulated the following recommendations:

1. Regular dermatological evaluations are imperative for individuals undergoing long-term HU therapy. We recommend implementing annual routine dermatological follow-ups for all patients undergoing long-term HU treatment, irrespective of whether they have presented with any skin lesions. Additionally, for patients who have developed precancerous or cancerous skin lesions, we advise dermatological assessments every three to six months, with the specific frequency determined by the dermatologist based on the extent and severity of the lesions.

2. Any suspicious skin lesions should be timely biopsied. Early diagnosis facilitates the selection of suitable treatments for these lesions, preventing their progression to an advanced stage. This proactive approach serves to reduce the morbidity and mortality associated with such conditions, minimize the utilization of healthcare resources, and mitigate the related financial burdens. 

3. A multidisciplinary collaboration between dermatologists and hematologists is highly needed. In certain instances, discontinuation of the responsible medication may not be a viable option. Consequently, a collaborative effort is essential to tailor treatment plans effectively and ensure the best course of action.

4. Continued patient monitoring is crucial even after discontinuing HU treatment. This necessity arises from the understanding that the skin-related toxicity caused by HU is an enduring cumulative process that persists following drug cessation, with the potential for NMSC recurrence several years later.

5. Patients should be provided with comprehensive information on all potential side effects, stressing the need to promptly report any significant and advancing skin reactions.

6. We recommend the consistent implementation of UV protection measures for all patients undergoing long-term therapy with HU.

7. It is highly necessary to conduct extensive prospective multi-center studies to unveil the incidence, morbidity, and mortality rates among patients developing NMSCs while undergoing HU treatment. There is still limited research available on this subject.

8. Randomized clinical trials need to be conducted to explore the potential benefits of using oral retinoids as a preventive measure against NMSCs in individuals undergoing HU treatment, especially in cases requiring prolonged therapy and presenting with skin abnormalities.

## Conclusions

This case study underscores the elevated risk of NMSCs in patients receiving long-term HU treatment. Our objective is to enhance vigilance in this regard and to provide clinicians, particularly from a dermatological standpoint, with valuable insights and recommendations to potentially mitigate the morbidity and mortality associated with these skin cancers.
